# Correction

**DOI:** 10.1111/cas.15643

**Published:** 2023-03-02

In Masaaki Kusunose et al. (2015),^1^ Figures 1(g), 2(f), S2(B) were published with incorrect images and Figure S2(e), S2(f), S2(g), and S2(h) with incorrect labels.

Figure 1(g) has been replaced by the correct image shown below.

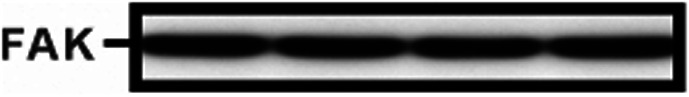

Figure 2(f) has been replaced by the correct image shown below.

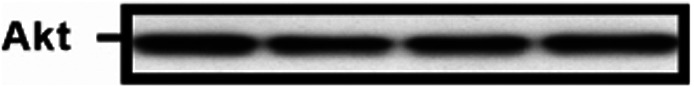

Figure S2(B) has been replaced by the correct image shown below.

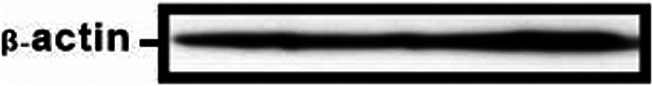

The label of “G4A Δ tail” in Figure S2(e), S2(f), S2(g), and S2(h) should be corrected to “G4A tail only”. Figure legend of S2 is correct and remained same.

The authors apologize for the errors. Masaaki Kusunose, Naozumi Hashimoto, Motohiro Kimura, Daisuke Aoyama, Koji Sakamoto, Shinichi Miyazaki, Akira Ando, Norihito Omote, Kazuyoshi Imaizumi, Tsutomu Kawabe, Yoshinori Hasegawa agree with this notice. Ryo Ogata disagrees with this notice.

This correction does not affect the results or conclusions drawn from the data in any way.
